# Unraveling the Role of EV-Derived miR-150-5p in Prostate Cancer Metastasis and Its Association with High-Grade Gleason Scores: Implications for Diagnosis

**DOI:** 10.3390/cancers15164148

**Published:** 2023-08-17

**Authors:** Marian Cruz-Burgos, Sergio A. Cortés-Ramírez, Alberto Losada-García, Miguel Morales-Pacheco, Eduardo Martínez-Martínez, Jorge Gustavo Morales-Montor, Alejandro Servín-Haddad, J. Samuel Izquierdo-Luna, Griselda Rodríguez-Martínez, María del Pilar Ramos-Godínez, Vanessa González-Covarrubias, Abraham Cañavera-Constantino, Imelda González-Ramírez, Boyang Su, Hon S. Leong, Mauricio Rodríguez-Dorantes

**Affiliations:** 1 Laboratorio de Oncogenómica, Instituto Nacional de Medicina Genómica (INMEGEN), Mexico City 14610, Mexico; marian.cruz.bqd14@outlook.com (M.C.-B.);; 2 Laboratory of Cell Communication and Extracellular Vesicles, Instituto Nacional de Medicina Genómica (INMEGEN), Mexico City 14610, Mexico; 3 Urology Department, Hospital General Dr. Manuel Gea Gonzalez, Mexico City 14080, Mexico; gmontorm@hotmail.com (J.G.M.-M.); haddad_ahs@hotmail.com (A.S.-H.); 4 Hospital Central Militar, Servicio de Urología, Mexico City 11600, Mexico; 5 Laboratorio de Microscopía Electrónica, Instituto Nacional de Cancerología, Mexico City 14080, Mexico; pilyrg@gmail.com; 6 Laboratorio de Farmacogenómica, Instituto Nacional de Medicina Genómica (INMEGEN), Mexico City 14610, Mexico; 7 Departamento de Anatomía Patológica, Centro Estatal de Oncología, Campeche 24090, Mexico; 8 Departamento de Atención a la Salud, Universidad Autónoma Metropolitana, Mexico City 14387, Mexico; 9 Department of Medical Biophysics, Temerty Faculty of Medicine, University of Toronto, Toronto, ON M5G 1L7, Canada; 10 Biological Sciences Platform, Sunybrook Research Institute, Toronto, ON M4N 3M5, Canada

**Keywords:** prostate cancer, metastasis, miRNA, lymph node, extracellular vesicles, exosome, biomarkers

## Abstract

**Simple Summary:**

This study focuses on prostate cancer metastasis, a leading cause of patient mortality. The research explores extracellular vesicle (EV)-enclosed microRNAs as potential diagnostic tool for prostate cancer. Using microarray analysis, the study identifies distinct microRNA expression profiles in metastatic lymph nodes, primary tumor tissues, and non-metastatic lymph nodes. Notably, miR-140-3p, miR-150-5p, and miR-23b-3p exhibit differential expression. Evaluation of exosomes from plasma and cancer cells reveals low miR-150-5p expression associated with high Gleason scores, suggesting its role in Wnt pathway regulation and bone metastasis. EV-derived miR-150-5p emerges as a promising diagnostic marker for early-stage metastasis and high-grade Gleason scores.

**Abstract:**

Metastasis remains the leading cause of mortality in prostate cancer patients. The presence of tumor cells in lymph nodes is an established prognostic indicator for several cancer types, such as melanoma, breast, oral, pancreatic, and cervical cancers. Emerging evidence highlights the role of microRNAs enclosed within extracellular vesicles as facilitators of molecular communication between tumors and metastatic sites in the lymph nodes. This study aims to investigate the potential diagnostic utility of EV-derived microRNAs in liquid biopsies for prostate cancer. By employing microarrays on paraffin-embedded samples, we characterized the microRNA expression profiles in metastatic lymph nodes, non-metastatic lymph nodes, and primary tumor tissues of prostate cancer. Differential expression of microRNAs was observed in metastatic lymph nodes compared to prostate tumors and non-metastatic lymph node tissues. Three microRNAs (miR-140-3p, miR-150-5p, and miR-23b-3p) were identified as differentially expressed between tissue and plasma samples. Furthermore, we evaluated the expression of these microRNAs in exosomes derived from prostate cancer cells and plasma samples. Intriguingly, high Gleason score samples exhibited the lowest expression of miR-150-5p compared to control samples. Pathway analysis suggested a potential regulatory role for miR-150-5p in the Wnt pathway and bone metastasis. Our findings suggest EV-derived miR-150-5p as a promising diagnostic marker for identifying patients with high-grade Gleason scores and detecting metastasis at an early stage.

## 1. Introduction

Prostate cancer (PCa) is a prevalent malignancy, ranking as the second most common cancer and the sixth leading cause of death in men worldwide [1]. In Mexico, PCa exhibits the highest incidence rate of all cancers among men over 50 years, with a rate of 29.9% and a mortality rate of 16.9% [1].

The gold standard for PCa diagnosis involves assessing levels of prostate-specific antigen (PSA) in the blood. However, this method’s limitations include a high rate of false positives and overdiagnosis, often leading to unnecessary overtreatment, as benign conditions like prostatic hyperplasia can cause elevated PSA levels [2]. Consequently, 76% of prostate biopsies conducted after elevated PSA levels (>3–4 ng/mL) fail to detect cancer [2,3]. As a result, PCa is frequently diagnosed at advanced stages, when surgical intervention is no longer feasible and high-dose androgen blockade therapy becomes the only treatment option [4]. Although PSA levels may initially decrease after treatment, they typically rise again within 12 to 18 months, marking the development of castration-resistant PCa (CRPC), which carries a significant risk of metastatic spread. High-risk Gleason scores (>7) are associated with 15% of newly diagnosed PCa patients with assessable pelvic lymph node metastasis, while 22% are at high risk of developing distant metastasis [5,6,7]. Bone metastasis occurs in approximately 10% of newly diagnosed cases, escalating to 80% in advanced stages, leading to a mere 28% 5-year survival rate for metastatic PCa patients [6,8,9,10]. Given the unclear mechanisms underlying PCa metastasis development, there is an urgent need to implement molecular tools for diagnosis and prevention to address these challenging statistics.

Lymph nodes (LNs) play a crucial role in enhancing tumor cell invasiveness and migration capacity. The interaction between tumor cells and resident immune cells within LNs promotes immunosuppression and immune tolerance, contributing to subsequent metastatic disease and poor outcomes in various tumor types [11,12,13,14]. Although the molecular distinctions between primary tumor cells and metastatic cells in LNs are not yet fully elucidated, the presence of tumor cells in the LNs serves as a critical prognostic indicator for metastasis in cancers such as melanoma, breast, oral, pancreatic, and cervical cancers. Consequently, the identification of a metastatic LN typically suggests the possibility of distant organ invasion [15,16]. The “seed and soil” hypothesis proposed by Stephen Paget suggests that tumor cells can only grow in organs or tissues providing the necessary conditions for their survival [17]. Previous research has demonstrated that metastatic cells originating from LNs can disseminate to distant organs, increasing circulating tumor cells and metastatic foci [18]. However, the metastatic potential of cancer cells derived from LNs remains a subject of ongoing debate, as factors enabling tumor cells’ survival in a foreign microenvironment may also facilitate their survival in distant organs [19]. 

Tumor cells secrete various factors that create a microenvironment, conducive to the formation of pre-metastatic niches (PMNs), where metastasis can occur. These factors include proteins, nucleic acids, and extracellular vesicles (EVs), which can be detected in body fluid like blood, plasma, urine, and saliva [20,21]. Among the diverse subtypes of EVs, exosomes stand out due to their involvement in intercellular communication and potential utility in liquid biopsies [22]. Exosomes, derived from endocytic processes, possess a lipid membrane structure and a spheroid shape, typically ranging in size from 30-200 nm. Their composition includes proteins, lipids, metabolites, and nucleic acids, including DNA, RNA, and microRNAs (miRNAs) [23]. Exosomal miRNAs have been implicated in various mechanisms associated with cancer and metastasis progression, making them potential predictors of metastasis when detected in tissue [24,25,26]. Additionally, exosomal miRNAs have been shown to reflect the pathological state of cells and modify cellular pathways, contributing to PMN formation [27,28,29,30,31,32]. Their stability in various sample types, including paraffin-embedded tissues and body fluids, makes miRNAs suitable diagnostic molecules [33,34,35]. In the context of PCa, miRNAs represent valuable biomarkers offering supplementary value to PSA in the diagnosis and prediction of disease progression. Unlike PSA, which lacks specificity to distinguish between benign conditions and cancer [36], specific miRNA expression ratios (miR-1913/miR-3659 and miR-H9/miR-3659) have shown promise in differentiating PCa patients from healthy individuals, particularly in the grey zone of PSA levels (3–10 ng/mL). Notably, miRNAs such as miR-21-5p, miR-574-3p, and miR-6880-5p have been associated with CRPC. Overall, miRNAs contribute additional value beyond PSA as biomarkers for PCa diagnosis and prediction of disease progression, given their involvement in regulating key factors implicated in PCa cell growth, castration resistance, the Notch signaling pathway, DNA damage, and apoptosis [37].

The objective of this study is to identify miRNAs with differential expression in metastatic lymph node tissue, confirm their presence in EVs in plasma, and determine any specific association between these miRNAs and risk groups of PCa patients based on Gleason scores. We aim to prove the potential role of these miRNAs as biomarkers for identifying patients with a higher risk of developing metastasis. This information can guide decisions on whether patients should undergo more aggressive interventions like lymphadenectomy and implementation of closer monitoring strategies, considering the close association between Gleason score and tumor aggressiveness.

In this study, we identified differential expression of miR-150-5p and miR-23b-3p levels between metastatic lymph nodes and both non-metastatic lymph nodes and primary tumor tissue in PCa samples. Subsequent validation of miRNA expression in EVs from plasma samples demonstrated statistically significant differences in miR-150-5p expression, particularly in samples with a Gleason score > 7 (high risk, PSA > 20 ng/mL, Gleason score > 7) [5]. We discuss our observations regarding the potential utility of miR-150-5p and miR-23b-3p as potential biomarkers for PCa metastasis risk stratification.

## 2. Materials and Methods

### 2.1. Tissue and Plasma Collection

Tissue and plasma samples were collected from PCa patients and healthy subjects. For microarray assays, paraffin tissue samples (*n* = 12) and plasma samples from PCa patients (*n* = 16) and healthy individuals (*n* = 10) were obtained from Dr. Alejandro Haddad Servin at Hospital General Manuel Gea González in Mexico City. The samples included metastatic lymph nodes, non-metastatic lymph nodes, and tumor tissues, collected based on pathology scoring criteria at the time of collection. The cohort comprised three paired metastatic lymph node–lymph node tissues and three paired non-metastatic lymph node–tumor tissues. For EVs assays, a new cohort of samples was used, consisting of plasma samples from PCa patients (*n* = 31) and healthy individuals (*n* = 12) provided by Dr. Hon Leong at Sunnybrook Research Institute in Toronto, Canada. The healthy donors were aged between 26 and 45 and with no history of PCa. Ethical approval for this study was obtained from the Research Ethics Board of Sunnybrook Health Sciences Centre, and all donors provided informed consent (identification number 5617). For late miRNA validation, PCa tissue samples (*n* = 15) were obtained from Dr. Abraham Cañavera at Centro Estatal de Oncología, Campeche, Mexico, and metastatic tissue samples (*n* = 5) were obtained from Dr. Gustavo Morales Montor from Hospital Angeles, Mexico City. Control tissues (*n* = 15) were acquired by INCIFO, Mexico, during forensic procedures from healthy males with no evident indication of PCa at the time of sample collection. The collection of all tissue and plasma samples in Mexico was approved by the Ethics Committee for Research at the National Institute of Genomic Medicine (identification number CEI 2020/30).

### 2.2. Cell Culture and Conditioned Media Collection

Human prostate carcinoma cell lines LNCaP, PC3, and DU145 were obtained from the American Tissue Culture Collection (ATCC, Inc., Manassas, VA, USA). BPH-1, NHPrE-1, and BHPrE-1 cells were kindly provided by Dr. Simon Hayward. Cells were cultured in RPMI 1640 or DMEM (Sigma-Aldrich, Inc., Saint Louis, MO, USA) supplemented with 5–10% fetal bovine serum (FBS) at 37 °C and a 5% CO_2_ atmosphere. For conditioned media collection, cells were grown in medium supplemented with exosome-depleted FBS. Exosome-depleted FBS was obtained by subjecting FBS to ultracentrifugation at 100,000× *g* for 18 h at 4 °C, discarding the pellet, and filtering the serum through a 0.25 µm filter. When cells reached 70–80% confluence, they were treated with RPMI 1640 or DMEM supplemented with exosome-depleted FBS for 72 h.

### 2.3. Microarrays from Paraffin Tissues and Plasma Samples

Total RNA from paraffin tissues was isolated using the RNeasy FFPE kit (Qiagen, Hilden, Germany) following the manufacturer’s instructions. The total RNA was quantified using NanoDrop One (Thermofisher Scientific, Inc., Waltham, MA, USA). For miRNA expression analysis, a standard protocol was followed. Briefly, 250 ng of total RNA from each sample was labeled using the FlashTag™ Biotin RNA Labeling kit (Affymetrix^®^; Thermo Fisher Scientific, Inc.), and the labeled RNA was hybridized with the GeneChip miRNA 4.0 Array (Affymetrix^®^; Thermo Fisher Scientific, Inc.). The miRNA microarray chips were washed twice with 1X PBST (0.02% Tween) buffer and stained with FlashTag™ Biotin HSR (Affymetrix; Thermo Fisher Scientific, Inc.) for 5 min at 35 °C. CEL files were generated after digitizing the image. The fluorescence intensity values in CEL format were pre-processed and normalized by quartiles using Expression Console™ v1.4.1.46 software (Affymetrix; Thermo Fisher Scientific, Inc.). Transcriptomic Analysis Console™ v4.0 software (Affymetrix; Thermo Fisher Scientific, Inc.) was used for expression analysis and heatmap generation, where the CHP files generated after normalization were loaded. Differentially expressed miRNAs were identified by ±1.5-fold change and eBayes *p*-values of < 0.05 between each sample group.

### 2.4. Ultracentrifugation for EV Isolation

Conditioned media (150 mL) were subjected to differential centrifugation at 400× *g* for 10 min at 4 °C, followed by 2000× *g* for 20 min at 4 °C. The supernatant was then ultracentrifuged at 110,000× *g* (37,000 rpm, k-factor 191) for 2 h at 4 °C using a 45Ti rotor (Beckman Coulter, Brea, CA, USA) in an Optima L-100 XP ultracentrifuge (Beckman Coulter). The resulting pellet, containing EVs, was resuspended in 2.8 mL of filtered PBS and subjected to another ultracentrifugation step at 110,000× *g* (32,500 rpm, k-factor 152) for 2 h at 4 °C using a SW55Ti rotor (Beckman Coulter). The pellet was then resuspended in 50 uL of PBS for electron microscopy or 100 uL of RIPA buffer (Sigma-Aldrich, Inc.) for protein extraction.

### 2.5. Aqueous Two-Phase Separation for EV Isolation

A solution of 20% dextran (Pharmacosmos, Berkshire, UK) and 40% polyethylene glycol (Sigma-Aldrich, Inc.) (ATPS mix) was prepared and mixed with 10 mL of conditioned media previously centrifuged at 2000× *g* for 20 min at 4 °C. For plasma EV isolation, plasma samples were centrifuged at 2000× *g* for 15 min at 4 °C, and 500 uL of the plasma was mixed with 4.5 mL of filtrated PBS. The diluted plasma was then mixed with the ATPS mix, and the resulting solution was centrifuged at 200× *g* for 25 min at 4 °C. The dextran phase, containing EVs, was collected and stored at −80 °C for nanoscale flow cytometry analysis. 

### 2.6. Western Blot for EV Markers

Cell protein extraction was performed with RIPA buffer (Sigma-Aldrich, Inc.) according to the manufacturer’s protocol. The EV proteins collected by ultracentrifugation were quantified using the DC protein assay (Bio-Rad, Hercules, CA, USA) following the manufacturer’s instructions. Western blotting was performed as follows: proteins were run on 12% polyacrylamide gels, and electrophoresis was carried out at 75 V for 20 min and then at 100 V for 120 min. The polyacrylamide gels were transferred to PVDF membranes using a semi-dry system at 15 V for 45 min. Membrane blocking was performed with 5% low-fat milk in TBST buffer overnight. After three washes with TBST-Tween 1%, primary antibodies were added to 1% low-fat milk overnight at 4 °C with agitation. The next day, the membrane was incubated with HRP-conjugated secondary antibodies in 1% low-fat milk for 2 h. After three washes with TBST, membrane images were obtained using the HRP system Luminata Forte (Merck, Darmstadt, Germany) for 5 min. The following antibodies were used: anti-CD9 (Santa Cruz, Dallas, TX, USA, cat. sc-13118), anti-CD63 (Thermo Fisher Scientific, Inc., cat. 10628D), anti-TSG101 (Abcam, Cambridge, UK, cat. cab83), anti-GAPDH (Abcam, cat. ab8245), anti-β-actin (Abcam, cat. ab8221), anti-rabbit HRP secondary (Abcam, cat. ab97051), and anti-mouse HRP secondary (Cell Signaling, Danvers, MA, USA, cat. 7076S).

### 2.7. Electron Microscopy for EVs

EVs isolated by ultracentrifugation were resuspended in 50 µL of 0.2 µm filtered PBS, and the following dilutions were used for each cell line: LNCaP 1:8, PC3 1:30, and DU145 1:15. The EVs were fixed with 2.5% glutaraldehyde–paraformaldehyde in 0.1 M sodium cacodylate buffer (Electron Microscopy Sciences, Hatfield, PA, USA) for 40 min. After incubation, 15 µL of the EV solution was placed on the top of a 200-mesh formvar/carbon-coated grid (Ted Pella, Inc., Redding, CA, USA) for 25 min. After three washes with distilled water, staining was performed with 4% alcoholic uranyl acetate for 7 min. Images were obtained using a JEOL JEM-1010 electron microscope equipped with an automated machine teller (AMT) digital camera.

### 2.8. Nanoscale Flow Cytometry

Fluorophore-coupled antibodies and an isotype control were incubated with 10 µL of ATPS EV samples (isolated from cell lines and plasma) for 30 min at 4 °C in the dark. The following antibodies were used: PE Mouse Anti-Human CD81 (BD Pharmigen, San Diego, CA, USA, cat. 561957), PE Mouse Anti-Human CD63 (BD Pharmigen, cat. 581925) PE Mouse Anti-Human CD9 (BD Pharmigen, cat. 555372), and PE Mouse IgG K Isotype Control (BD Pharmigen, cat. 556650). After incubation, the samples were diluted in 900 µL of 0.02 µm filtered PBS and analyzed using the A50-Micro Nanoscale Flow Cytometer (Apogee FlowSystems Inc., Hertfordshire, UK) to detect CD9-, CD63-, and CD81-positive EVs with a diameter of <200 nm. The nanoscale flow cytometer was calibrated following the protocol described in https://doi.org/10.18632/oncotarget.26620 (accessed on 5 August 2022). Manual gating based on the size of the population of interest was performed using Apogee Histogram Software v6.0.70. The data were exported to Excel, and the total number of events that occurred within the gate was used for statistical analysis in GraphPad Prism 8.

### 2.9. RT-qPCR for miRNA Validation

RNA from cells and EVs from cell lines and fresh tissues were isolated using the Trizol method. RNA from EVs in plasma samples was isolated using the exoRNeasy kit (Qiagen), following the manufacturer’s instructions with some modifications. Briefly, the dextran phase from ATPS isolation was mixed with XBP buffer and bound to the column, and the subsequent steps were performed according to the manufacturer’s instructions. Paraffin tissue samples were isolated using the RNeasy FFPE kit (Qiagen). Retrotranscription was performed using the TaqMan MicroRNA Reverse Transcription Kit (Thermo Fisher Scientific Inc.) according to the manufacturer’s instructions. For real-time PCR, the following TaqMan probes were used: hsa-miR-23b-3p (Thermo Fisher Scientific Inc., cat. 245306_mat), hsa-miR-150-5p (Thermo Fisher Scientific Inc., cat. 000473), and RNU6B (Thermo Fisher Scientific Inc., cat. 001093).

### 2.10. Prediction of Target Genes and Cellular Pathways of Differentially Expressed miRNAs

To identify the target genes of the differentially expressed miRNAs, we used three different open-access databases: TargetScan v8.0, miRTarBase v9.0, and Diana microT-CDS v5.0. Predicted miRNA–mRNA interactions were reported by TargetScan v8.0 and microT-CDS v5.0, while miRTarBase reported validated targets. To ensure accuracy, we selected the targets that were identified in at least two of these databases. The identified target genes were then used as input for the Metascape platform (https://metascape.org/gp/index.html#/main/step1 (accessed on 5 August 2022)) to perform functional gene annotation using Gene Ontology (GO) terms and Kyoto Encyclopedia of Genes and Genomes (KEGG) pathway enrichment analysis. Enriched pathways with *p*-values lower than 0.05 were considered significantly enriched.

## 3. Results

### 3.1. Differentially Expressed miRNAs in Metastatic Lymph Nodes

To investigate the presence of a miRNA signature in metastatic lymph nodes, we collected paraffin-embedded tissues from patients with PCa, including metastatic lymph nodes (MLN), non-metastatic lymph nodes (LN), and primary tumors (T). Clinical information for the study population is summarized in Table 1.

**Figure 1:**
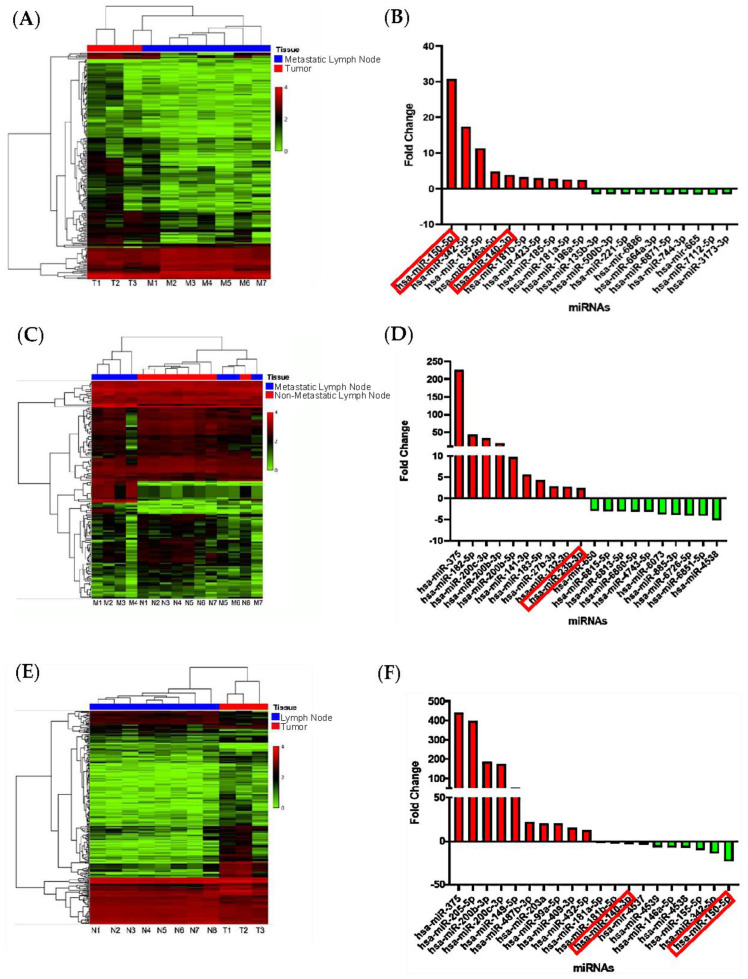
Comparative analysis of miRNA expression in metastatic lymph nodes, PCa tumors, and non−metastatic lymph nodes. Heatmaps depict miRNA expression profiles, with red indicating increased expression and green indicating reduced expression. The bar graph displays the top 20 miRNAs with the highest fold change value and *p* < 0.05. (**A**,**B**) miRNA expression comparison between metastatic lymph node and PCa tumor. (**C**,**D**) Comparison of metastatic lymph node tissue and non-metastatic lymph node. (**E**,**F**) Comparison of tumor and non-metastatic lymph node. Fold change ±1.5, *p* < 0.05, eBayes ANOVA.

Bioinformatic analysis of microarray data revealed distinct miRNA expression patterns in the different tissue comparisons. Comparing MLN to T samples, we identified 15 upregulated and 142 downregulated miRNAs (Figure 1A). Similarly, in the comparison of MLN versus LN, we found 21 upregulated and 91 downregulated (Figure 1C). The T versus LN comparison showed 144 upregulated and 42 downregulated miRNAs (Figure 1E). Figure 1B,D,F show the top 20 miRNAs with the highest fold change and *p*-value < 0.05 for each comparison. For a comprehensive list of differentially expressed miRNAs in each comparison, refer to Tables S1–S3. Among these miRNAs, 43 were differentially expressed across the comparisons, with 17 miRNAs shared among the different tissues. Further investigation and validation of these 43 unique miRNAs were carried out in plasma samples. Our findings provide novel evidence of distinct miRNA expression patterns between MLN and tumor tissue samples, with most miRNAs showing downregulation in metastatic tissue.

### 3.2. miRNAs from Metastatic Lymph Nodes Are Found in Plasma of PCa Patients

**Figure 2:**
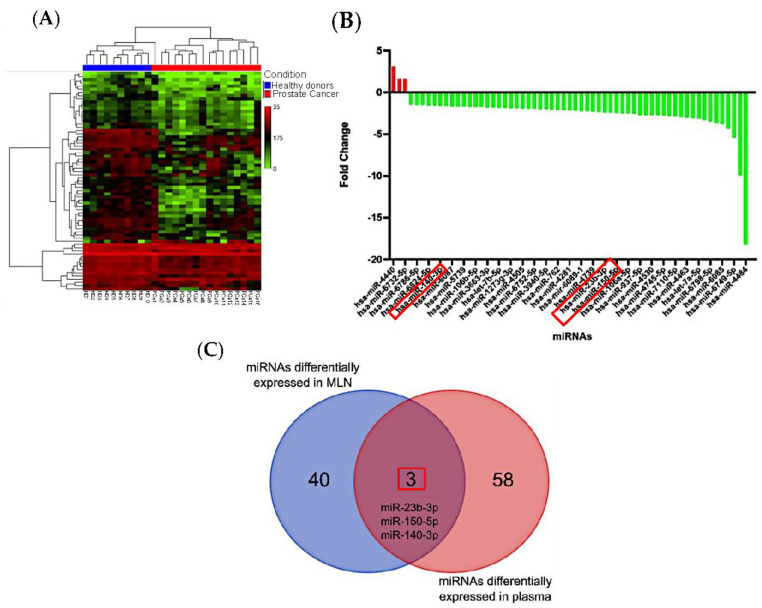
Comparative analysis of miRNA expression in plasma from PCa patients and healthy individuals. (**A**) Heatmaps depicting the expression profiles of miRNAs, with red indicating increased expression and green indicating reduced expression. (**B**) The bar graph displays the differentially expressed miRNAs in PCa plasma based on a *p*-value < 0.05 and a fold change of 1.5. (**C**) The Venn diagram illustrating the overlapping miRNAs differentially expressed in both paraffin-embedded tissues and plasma samples. Three miRNAs, namely miR−140−3p, miR−23b−3p, and miR−150−5p, were found to be present in both sample types.

To investigate whether the differentially expressed miRNAs identified in MLN are detectable in plasma, we conducted a microarray analysis on total RNA extracted from plasma samples of PCa patients and healthy donors. The discovery cohort included 26 samples, comprising 16 from PCa patients and 10 from healthy donors (Table 2). A total of 61 differentially expressed miRNAs were detected in PCa plasma samples compared to healthy subjects (Figure 2A). Refer to Table S4 for the complete list of differentially expressed miRNAs. 

Among these differentially expressed miRNAs, only three were found to be upregulated in PCa plasma samples (Figure 2B). The full list of differentially expressed miRNAs is provided in Table S5. Interestingly, three miRNAs (miR-140-3p, miR-150-5p, and miR-23b-3p) showed differential metastatic tissue and PCa plasma samples (Figure 2C). However, it is noteworthy that the expression patterns of these shared miRNAs were the opposite: upregulated in tissue and downregulated in plasma (Table 3). Based on the highest expression changes and lowest *p*-values, miR-150-5p and miR-23b-3p were selected for further evaluation of their presence in EVs in subsequent experiments (Table 3). MiR-140-3p was not chosen due to its less substantial expression changes compared to miR-150-5p and miR-23b-3p.

The results demonstrate the feasibility of detecting differentially expressed miRNAs from metastatic lymph node tissue in PCa plasma samples. To explore the possibility of miRNAs playing a role in intercellular communication, we further investigated the presence of miR-150-5p and miR-23b-3p in EVs. 

It is crucial to acknowledge the main limitations of this experiment. The microarray result showed a non-significant difference in expression levels of miR-150-5p between MLN and LN, as indicated by the statistical analysis (Tables S1–S3). Additionally, most of the detected miRNAs, including the selected miR-150-5p and miR-23b-3p, did not reach the conventional significance threshold of adjusted *p*-values < 0.05, but they showed a trend toward significance with *p* < 0.05. Despite these limitations, it is noteworthy that miR-150-5p exhibited upregulation in MLN compared to T, suggesting a potential role in the metastatic process. Its relevance as a biomarker may extend beyond discriminating between MLN and LN, encompassing specific pathways or interactions within the tumor microenvironment. Considering these factors, we have chosen miR-150-5p as our primary focus for further investigation into the molecular changes underlying tumor cell metastasis and survival within the lymph node. Therefore, we intend to include this miRNA in future validation studies.

### 3.3. Validation of EVs Isolated from Conditioned Media

To confirm the successful isolation of EVs from PCa cell lines (LNCaP, PC3, and DU145), we compared two different methods: ultracentrifugation and an aqueous two-phase system (ATPS). Transmission electron microscopy (TEM) analysis of EVs from ultracentrifugation confirmed the presence of EVs with varying sizes and shapes. The size of the isolated EVs matched the reported size for exosomes (less than 200 nm) (Figure S1). Western blot analysis revealed that EVs from PC3 and LNCaP cells contained the exosome markers TSG101, CD9, and CD63. EVs from DU145 cells exhibited a faint CD9 band and tested negative for GAPDH and β-actin, suggesting low levels of these markers in their vesicles (Figure S2). For practical purposes, vesicles smaller than 200 nm in size and positive for exosomal markers (CD9, CD63, CD81, and TSG101) will be considered exosomes.

In the case of ATPS, we confirmed the presence of EVs using nanoscale flow cytometry to measure particle size (<200 nm) and assess positivity for specific tetraspanin antibodies (CD9, CD63, and CD81) (Figure S3). A comparison between EVs isolated by ATPS and ultracentrifugation demonstrated a higher number of EVs smaller than 200 nm and positive for tetraspanins (potential exosomes) with ATPS (Figure S4). Interestingly, significant differences in total EVs and exosomes, as indicated by all the antibodies, were observed in DU145 (Figure S4).

The results from the analysis of membrane protein markers suggest that ATPS is a more sensitive technique than ultracentrifugation for isolating exosomes, as it enables their enrichment during the isolation process. 

### 3.4. miRNAs Are Enriched in EVs from Tumoral and Non-Tumoral PCa Cell Lines

After isolating EVs, we assessed the expression of miR-150-5p and miR-23b-3p in EVs derived from these cell lines. Additionally, three non-tumoral cell lines (BPH-1, BHPrE-1, and NHPrE-1) were included to compare the expression of EVs from both tumoral and non-tumoral cell lines.

**Figure 3:**
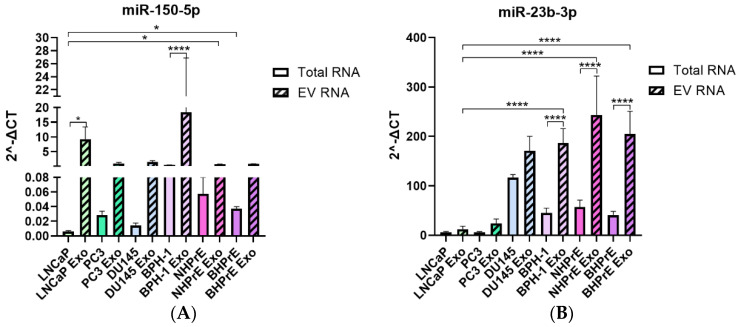
Comparative analysis of miRNA expression in EVs from tumoral and non-tumoral PCa cell lines. (**A**) Bar graph depicting the expression levels of miR-150-5p. EVs from LNCaP and BPH-1 exhibited the highest levels of miR-150-5p. (**B**) Bar graph illustrating the expression of miR-23b-3p. EVs from all non-tumoral cells demonstrated a higher enrichment of miR-23-3p. * *p* < 0.05, **** *p* < 0.0001.

Both miR-150-5p and miR-23b-3p, which were differentially expressed in paraffin tissues and plasma samples, were enriched in EVs from all cell lines, regardless of tumoral or non-tumoral origin (Figure 3). LNCaP and BPH-1 exhibited the highest levels of miR-150-5p (Figure 3A), whereas non-tumoral cell lines demonstrated higher enrichment of miR-23b-3p compared to LNCaP and PC3. DU145 showed similar enrichment of miR-23b-3p in both cells and EVs (Figure 3B).

These results suggest that the enrichment of miRNAs in EVs may be dependent on the cell type. Furthermore, our study reveals that EVs derived from non-tumoral cells have the potential to be enriched with specific miRNAs, such as miR-23b-3p, compared to tumor cells. This observation proposes selective packaging of miRNAs in EVs.

### 3.5. MiR-150-5p Is Associated with a High Gleason Score

**Figure 4:**
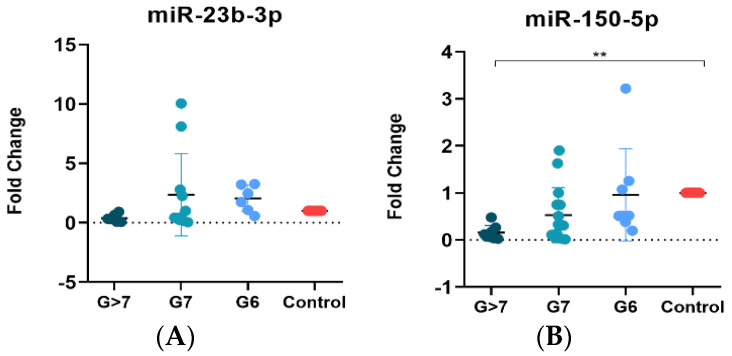
Expression of miR−150−5p and miR−23b−3p in plasma EVs. (**A**) Non-significant association between miR−23b−3p expression and Gleason stages, but reduced expression of miR−23b−3p with high Gleason score > 7. (**B**) Significant reduced expression of miR−150−5p in plasma EVs from samples with Gleason > 7. ** *p* < 0.01.

To investigate the potential of liquid biopsy-based miRNAs as predictors of PCa, we determined the expression of miRNAs in plasma exosomes in a new cohort of samples. These samples were divided into four groups: samples without PCa (control, *n* = 12), samples with Gleason score 6 (*n* = 8), samples with Gleason score 7 (*n* = 15), and samples with Gleason score > 7 (*n* = 8). Patients with PSA > 20 ng/mL or a Gleason score > 7 were considered high-risk patients according to EAU guidelines [5]. Clinical data for the samples are provided in Table 4. EVs in plasma samples from both healthy subjects and PCa patients were obtained using ATPS and analyzed by nanoscale flow cytometry (Figure S5). To demonstrate the isolation of exosomes and the presence of EVs, we decided to present the expression data of exosome-specific markers (CD9, CD63, and CD81). The population of EVs observed in plasma was lower than that observed in cell lines, and only CD9 was abundantly expressed, while CD63 and CD81 were virtually absent in plasma samples (Figure S7). Interestingly, the percentage of CD9 was lower in the samples with a Gleason score > 7 (Figure S6). Though no significant association for miR-23b-3p was found, its expression decreased in the Gleason score > 7 sample group (Figure 4A). In contrast, statistically significant differences were observed in the expression of miR-150-5p between the Gleason score > 7 sample group and the control samples, with a downregulation of the miRNA in the higher Gleason score group (>7) (Figure 4B).

**Figure 5:**
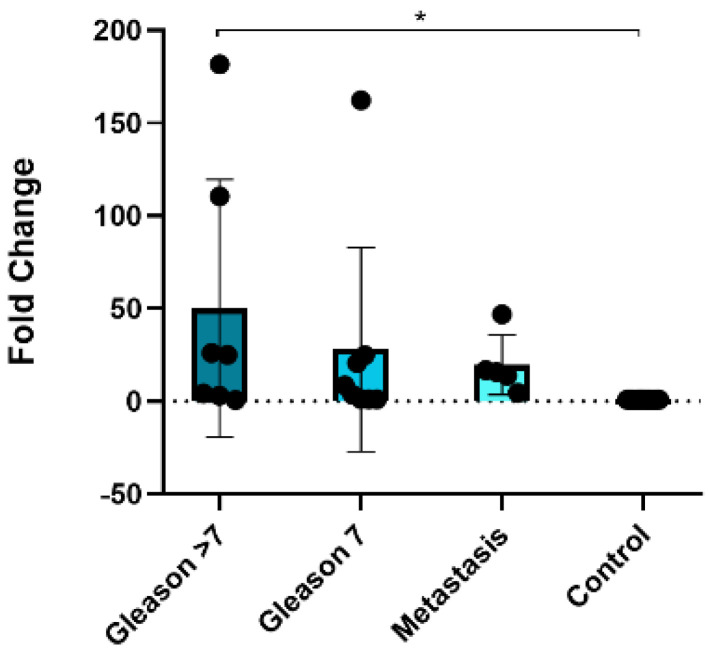
Validation of miR−150−5p expression in a new cohort of PCa, metastatic, and control tissues. Bar graph showing overexpression of miR−150−5p in Gleason > 7 tissues compared to control samples. * *p* < 0.05.

Despite the lack of significant changes, the fold change enrichment of miR-23b-3p was nearly five times greater than that of miR-150-5p. Therefore, we validated the expression of miR-150-5p in a new cohort of tissues including samples of primary tumor Gleason 7 (*n* = 7), Gleason < 7 (*n* = 7), among which two of these high-risk samples presented bone and lymph node metastasis, while others presented metastatic lymph nodes (*n* = 5) and normal prostate tissue (*n* = 20). The validation was carried out to confirm if our findings match up with those observed in plasma EVs. Clinical data of the samples are presented in Table 5. We found that miR-150-5p was overexpressed in PCa tissues with nodular metastasis compared to the control, consistent with our initial microarray results (Figure 5).

The direct association of exosomal miR-150-5p with a Gleason score > 7 in both plasma EVs and tissue samples, compared to control samples, suggests its potential as a diagnostic aid for high-risk PCa patients. Additionally, these findings indicate selective packaging of miR-150-5p in EVs depending on the disease state, suggesting its possible involvement in tumor progression and metastasis.

### 3.6. MiR-150-5p Is Potentially Associated with Bone Metastasis

**Figure 6:**
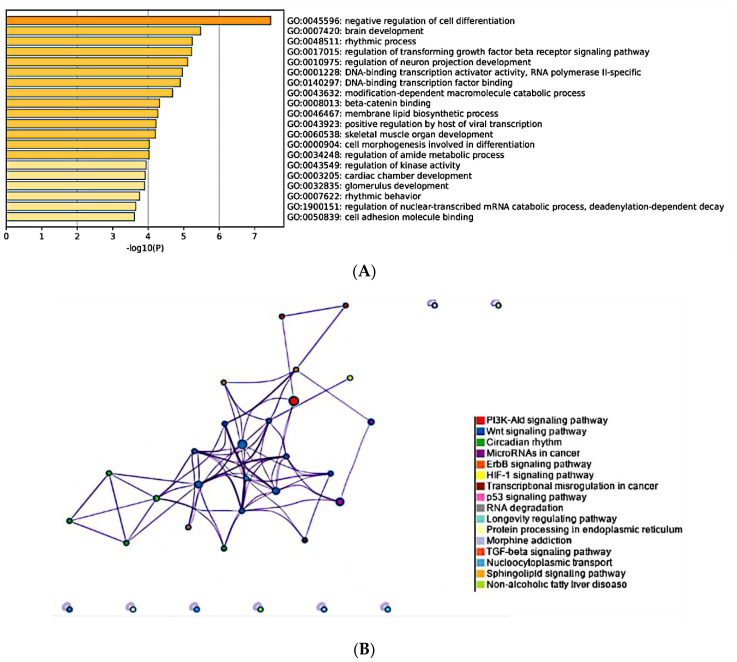
Pathway overrepresentation of miR−150−5p targets. (**A**) Gene Ontology analysis. Each bar represents the top twenty enriched GO terms, with the color of each bar representing the *p*-value for each term. (**B**) Network of enriched KEGG pathways. Each term is represented by a circle node whose size is proportional to the number of input genes that fall under that term, and its color represents its cluster identity. Terms with a similarity score > 3 are linked by an edge; the thickness of the edge represents the similarity score. Both GO and KEGG analysis had a *p*-value cutoff <0.05.

To elucidate the biological relevance of miR-150-5p, the only miRNA significantly different in the group of samples with a Gleason score > 7 compared to the control, we performed a pathway overrepresentation and Gene Ontology (GO) analysis for its targets. To ensure robustness, we employed information from three different databases (TargetScan, miRTarBase, microTCDS) for target prediction. Our analysis identified 139 potential targets for miR-150-5p. The GO analysis revealed that the most enriched GO term was negative regulation of cell differentiation (Figure 6A). Additionally, the targets were associated with important processes such as TGF-β signaling, β-catenin binding, cell morphogenesis involved in differentiation, skeletal muscle development, and cell adhesion molecule binding. Furthermore, the Kyoto Encyclopedia of Genes and Genomes (KEGG) pathway enrichment analysis identified several overrepresented pathways associated with miR-150-5p targets. The most prominent pathways included PI3K-Akt, Wnt, and circadian rhythm. Other significant pathways included ErbB, HIF-1, and p53 (Figure 6B).

The target genes associated with each GO term can be found in Table S6. These findings shed light on the possible involvement of miR-150-5p in critical cellular processes and signaling pathways, indicating its potential role in bone metastasis and tumor progression. Further investigation of these target genes and pathways may offer valuable insights into the molecular mechanisms underlying high-risk PCa and bone metastasis.

## 4. Discussion

In this study, we have provided evidence of distinct miRNA expression profiles in metastatic lymph nodes compared to non-metastatic lymph nodes and primary tumors in PCa. The role and mechanistic relevance of lymph node metastasis in PCa have not been extensively studied, and there is an urgent need for reliable biomarkers for early detection of metastasis. Previous research has demonstrated that lymphatic metastasis can serve as an indicator of prognosis and stage classification in breast and lung cancer [38,39]. Similarly, in PCa, metastatic lymph nodes have been associated with systemic disease and distant organ metastasis [40]. It has been proposed that tumor cells within the LNs can develop secondary metastases in remote locations. Furthermore, evidence supports the possibility of metastatic cell migration from the lymph node to distant organs [18,41]. These findings underscore the significance of lymph nodes as critical sites for tumor cell dissemination, emphasizing the need to target and understand this process for effective cancer management and treatment.

Out study revealed a nearly 30-fold higher expression of miR-150-5p in metastatic lymph nodes compared to tumor tissue, whereas it was underexpressed by 20-fold in plasma from PCa patients compared to healthy individuals. Moreover, miRNAs expressed in the tissue were generally downregulated in plasma samples, indicating discrepancies in miRNA expression across different sample types. Similar results have been reported in studies on rectal and breast cancer [42,43,44,45,46], suggesting that the inverse relationship in miRNA expression between different tissues is not specific to PCa. However, the mechanisms behind the reduction of miRNAs in plasma are not fully understood. Studies suggest that tumor cells may retain exocrine miRNA secretion, leading to a reduction in miRNA quantity in circulation, particularly in the more advanced metastatic stages [43]. 

Our results suggest that differential miRNA expression may indicate an adaptation of metastatic cells in the LNs, demonstrating distinctions from those in the primary tumor.

Previous studies in breast cancer have demonstrated that tumor cells evade the immune system in LNs by binding to immune cell receptors and creating a pro-tumoral microenvironment [47,48]. The alteration of the microenvironment in the LNs is associated with the concept of pre-metastatic niches (PMNs) [21], where the tumor releases factors that modify the target organ to support the survival and growth of scattered tumor cells. Animal models support the idea that distant metastasis occurs when cancer cells disseminate from the sentinel lymph nodes, promoting migration, extravasation, and invasion [18]. 

Tumor-derived EVs possess the ability to transport tumor cell-specific proteins and genetic material, including mRNA, miRNA, lnRNA, and dsDNA. The cargo within these EVs can subsequently be transferred to recipient cells residing in PMNs, either through paracrine signaling or systemic dissemination [20,49]. This transfer of EV content plays a crucial role in mediating diverse processes within the microenvironment, including the reprogramming of fibroblasts, specific targeting of immune cells, or modulation of the immune microenvironment in LNs [50,51,52]. Notably, research has shown that miRNAs expressed and secreted within EVs can impact vascular permeability [24,30], and the involvement of lymphatic vessels in transporting EVs has been demonstrated in studies on melanoma [53]. These findings highlight the crucial role of lymph nodes in facilitating the transportation of EVs and emphasize how changes in the LN microenvironment, mediated by miRNAs, can profoundly influence the development of metastasis. 

In our study, we chose to use total plasma to assess the overall expression profile of miRNAs. The comparison of miRNAs between tissue and plasma samples revealed the presence of miR-140-3p, miR-150-5p, and miR-23b-3p in both sample types. Among these, miR-150-5p and miR-23b-3p were selected for validation in EVs based on their higher fold change values in tissue comparisons, indicating their potential biological relevance and clinical impact. 

Although we found that miR-150-5p and miR-23b-3p were underexpressed in total plasma, we decided to assess their expression within EVs. Our main goal was to determine if these miRNAs were present inside the vesicles and to understand if their behavior differed inside and outside the vesicles compared to control samples. Additionally, EVs provide protection against RNases and offer information about their specific origin. miRNAs within EVs could potentially serve as plasma biomarkers while also providing valuable insights into their role in metastatic progression. The transfer of EVs between different cell types suggests the potential for paracrine miRNA action facilitated by this transportation. It is plausible that miRNAs could be selectively packaged into EVs and play a part in the formation of PMNs; however, we did not evaluate this possibility in our study. Nevertheless, this observation could set the stage for future investigations in this direction. 

To validate the successful isolation of EVs, we analyzed them using nanoscale flow cytometry to confirm the presence of tetraspanins and EVs smaller than 200 nm. Tetraspanins, including CD63, CD9, and CD81, are transmembrane molecules commonly associated with endosomes and enriched in EVs, making them reliable exosome markers [54,55]. The ATPS method resulted in a higher yield of EVs, including exosomes, compared to ultracentrifugation. Notably, the detection of tetraspanins in DU145 cells was only possible with ATPS, indicating the superior performance of this method (Figure 3 and Figure S2). Several factors related to the isolation technique may contribute to this difference. Firstly, ATPS yielded a higher quantity of vesicles compared to ultracentrifugation (Figure S4), leading to a higher amount of proteins in the sample [56]. Additionally, the high centrifugal force used in ultracentrifugation can damage vesicle structure, making it challenging for proteins to be recognized by Western blotting. In contrast, ATPS preserves the vesicular structures, enabling intact analysis of EVs by flow cytometry [57]. Moreover, ATPS enriches the population of tetraspanin-positive EVs, indicating a higher presence of these specific proteins within the vesicles. It is important to note that the frequency of canonical exosome markers may vary depending on the source of EVs, such as conditioned medium or biofluids from humans, and the choice of isolation method can significantly enrich specific markers in exosomes [58].

After validating the presence of EVs, we examined the expression of miRNAs in EVs from different cell lines. Our findings demonstrated that while miRNAs were generally enriched in EVs across all cell lines, specific miRNAs such as miR-150-5p showed significant enrichment in LNCaP and BPH-1 EVs, while miR-23b-3p exhibited higher enrichment in non-tumoral cell line EVs, including DU145 EVs. These results suggest that miRNA expression can vary depending on the cell type. The precise mechanisms underlying the selective packaging of miRNAs into EVs are not fully understood; however, studies indicate that proteins such as hnRN-PA2B and SYNCRIP can recognize specific motifs such as GGAG/UGCA and AGG/UAG, and GGCU, respectively, within the miRNA sequence and may be involved in targeting miRNAs for loading into EVs or retaining them within the cell [59,60]. Therefore, it would be intriguing to investigate whether miR-150-5p possesses any specific sequence that promotes its packaging into vesicles released by PCa cells.

While PSA remains the primary biomarker in blood for PCa diagnosis, its utility is limited due to high rates of false positives [2,3]. The identification of reliable and non-invasive biomarkers for PCa diagnosis and prognosis prediction is challenging, and the late detection of metastasis is a primary cause of death in PCa patients [61]. Plasma-free miRNAs have shown promise as diagnostic tools, but exosomal miRNAs, protected by a lipid bilayer membrane, are more stable and have been implicated in metastatic progression due to their role in intercellular communication [62]. Furthermore, miRNA levels in exosomes can be altered under different pathological conditions, including cancer [63,64,65].

The detection of miRNAs within EVs offers additional benefits compared to the detection of free miRNAs in plasma. EVs can contain a higher concentration of miRNAs than free miRNAs in plasma, which is particularly valuable when working with limited samples [66,67]. Moreover, EVs, including exosomes, play a crucial role in intercellular communication and the transfer of molecules such as proteins or nucleic acids, providing a more comprehensive understanding of the interactions between tumor cells and their micro- and macroenvironments. This can yield valuable information about the development of PMNs and real-time tumor evolution.

EVs in plasma derived from PCa patients were evaluated to search for potential miRNA biomarkers. To confirm the presence of EVs in plasma samples, flow cytometry analysis was performed. We found low levels of the CD9 marker in plasma EVs of Gleason > 7 samples. Additionally, higher levels of CD9-positive EVs were found in the control group compared to Gleason 7 and Gleason 6 (Figure S6). However, the absence or low abundance of other markers should not be interpreted as the absence of EVs, as tetraspanins can be present in low quantities in biological samples [58].

The expression of miR-23b-3p in Gleason 7 plasma samples was higher compared to the control group, but the observed differences were not statistically significant. In contrast, miR-150-5p showed significant differences in samples with a Gleason score > 7 compared to the control group, with the lowest expression of miR-150-5p in Gleason score > 7. The expression of CD9 shows a similar pattern to the expression of miRNAs (miR-23b-3p and miR-150-5p), supporting the idea that the miRNAs are indeed found inside the vesicles. In our final validation, miR-150-5p expression was higher in tumor tissue with Gleason > 7 compared to control samples, suggesting that this miRNA is closely related to the Gleason stage in the tumor. The expression of miR-150-5p was low in EVs and high in tissues, suggesting discrepancies in miRNA expression profiles. This finding is consistent with previous reports and may potentially be attributed to a selective mechanism governing the packaging of miRNAs during cancer progression [68,69]. Nevertheless, this study found that miR-150-5p could be a valuable biomarker, as it was associated with patients exhibiting a Gleason grade > 7 in plasma EVs. The significant reduction in miR-150-5p expression in plasma EVs from PCa patients with high Gleason scores compared to the control group has important implications for clinical management. The decreased miRNA expression could serve as an additional prognostic marker, aiding in the identification of patients with a higher risk of disease progression to metastasis or recurrence [70]. Furthermore, a comprehensive understanding of the miRNA expression profile in plasma may enable personalized treatment strategies, facilitating the development of targeted therapies based on specific miRNA alterations. Lastly, monitoring changes in miRNA expression over time in plasma samples may provide valuable insights into disease progression, treatment response, and the emergence of treatment resistance [71]. However, further validation through rigorous clinical studies involving larger patient cohorts is necessary to establish the clinical utility of plasma EV-based miRNA analysis in the management of PCa.

It is important to note that this study does not include a cohort of samples with BPH, which could limit the distinction between BPH and PCa patients with high levels of PSA [72]. Figure 3A shows elevated expression of miR-150-5p in both tumor cell-derived EVs and BPH EVs, suggesting a similar behavior in tissues. However, the miR-150-5p could be valuable in patients who have already received a PCa diagnosis but are unable to undergo a biopsy due to contraindications.

Finally, to elucidate the functional role of miR-150-5p, we conducted GO and KEGG pathway analyses. The analyses revealed that the target genes of miR-150-5p possibly regulate major pathways, including PI3-Akt, Wnt, ErbB, HIF-1, p53, and TGF-β pathways. The Wnt pathway has been associated with bone metastasis development, as evidenced by the GO terms β-catenin binding and skeletal muscle organ development, which were shared by the BCL9L target gene of miR-150-5p. Evidence shows its involvement in promoting mammary tumor growth and metastasis in mice as well as its activation in hepatocellular carcinoma under hypoxic conditions, both through BCL9/BCL9L and Wnt pathway activation [73,74]. 

In breast cancer, the inhibition of the Wnt pathway has been associated with enhancing osteoclast activity while inhibiting osteoblast formation and differentiation, directly contributing to bone metastasis [75]. In colorectal cancer (CRC), activated Wnt signaling transactivates miR-150-5p, leading to increased expression levels. This upregulation targets CREB, resulting in the downregulation of E-cadherin and ZO-1, promoting epithelial–mesenchymal transition (EMT) and enhancing cellular invasion and migration. Furthermore, the involvement of miR-150-5p in promoting metastatic processes in non-small cell lung cancer by targeting FOXO4 has been proven. The overexpression of miR-150-5p enhanced migration and EMT in cells, as evidenced by increased expression of N-cadherin, vimentin, NF-KB, and snail [76]. These findings highlight the critical role of the Wnt pathway in bone metastasis and suggest that miR-150-5p may regulate this pathway through its target genes, including BCL9L.

According to available evidence, miR-150-5p plays a significant role in the promotion of various types of cancer, particularly lung cancer. Increased levels of miR-150-5p in EVs and a simultaneous decrease in the expression of CD226 in natural killer (NK) cells have been observed under hypoxic conditions. The NK cells secrete immunosuppressive factors such as S100A8, and the upregulation of inflammatory and angiogenesis factors, including VEGF, CXCL1, CXCL2, and CXCL8, occurs. These cellular changes contribute to the enhanced metastasis and formation of tumor nodules in the lungs, as demonstrated in in vivo experiments; on the other hand, the knockdown of miR-150-5p leads to reduced cancer development and restoration of CD226 expression, thereby presenting a potential therapeutic avenue [77]. In contrast, individuals diagnosed with CRC display lower levels of exosomal miR-150-5p compared to healthy individuals, indicating the potential of miR-150-5p as a precise marker for identifying CRC patients [78]. A separate investigation on CRC has demonstrated that inhibition of miR-150-5p leads to increased cell viability and augmented expression of β-catenin in SW480 and HT-29 cells, ultimately fostering enhanced colony formation. These findings suggest miR-150-5p may play a tumor-suppressive role in colorectal cancer [79]. Importantly, in this study, the expression of miR-150-5p has been detected in both tissue and plasma samples obtained from PCa patients. MiR-150-5p has been linked to significant changes in prostatic tissue and has been associated with alterations in proliferation and invasion processes [80,81]. This study emphasizes the importance of miR-150-5p in the context of various cancers, highlighting its potential as a diagnostic marker and a target for therapeutic intervention. It is crucial to underscore that the small cohort sizes used in this study impose significant limitations on the obtained results, leading to a lack of statistical power. However, the present pilot study aims to explore the utilization of the lymph node as a source of data to identify miRNAs that play a role in the development of metastasis. It is important to highlight that the final decision regarding lymphadenectomy entirely depends on the urologist, even if the patient does not meet the Partin–Briganti criteria. Therefore, the expression of the miRNA could serve as a useful parameter for the urologist when making the decision about lymphadenectomy. Subsequently, these miRNAs can be employed as biomarkers. Here, we have successfully demonstrated the feasibility of detecting tissue-expressed miRNAs not only in plasma but also in extracellular vesicles, offering the potential to obviate the necessity for invasive biopsies. The role of EV-miRNAs in other cancer types has been proven. Under hypoxic conditions, tumor cells release EV-miR-301a-3p, which leads to polarization of tumor-associated macrophages, a contributing factor to the metastatic process in pancreatic cancer [82]. Furthermore, lower expression levels of miR-363-5p in plasma exosomes were significantly associated with breast cancer patients exhibiting lymph node metastasis [83].

According to the evidence, miRNAs contained in EVs (including miR-150-5p) play a crucial role in modifying the tumor microenvironment and can be transported to the PMN via EVs secreted by tumor cells. Nevertheless, further research is needed to fully comprehend their interplay in PMN formation.

These findings highlight the importance of early detection of this disease and the potential of metastatic lymph node tissue as a valuable resource for liquid biopsy development.

## 5. Conclusions

Metastatic lymph nodes exhibit distinct miRNA expression patterns compared to primary tumor tissue. These miRNAs are detectable in both cell and plasma EVs, with higher expression of miR-150-5p observed in plasma EVs from samples with a Gleason score > 7. Pathway analyses suggest that miR-150-5p may play a role in regulating the Wnt pathway. However, to fully validate the potential impact of miR-150-5p on tumor progression and metastasis, further research involving larger sample sizes and functional assays are needed.

## Figures and Tables

**Table 1:** Clinical data from the paraffin-embedded samples used in the microarray analysis.

Total of Samples	Metastatic Lymph Node		Non-Metastatic Lymph Node		Tumor	
	*n* = 7		*n* = 8		*n* = 3	
Age						
Mean	63.71		65.125		67.67	
Range	57–71		52–71		62–71	
PSA (ng/mL)						
Mean	15.64		13.06		18.18	
Range	8.35–27		8.13–18.11		9.43–27	
Gleason Score	*n*	%	*n*	%	*n*	%
3 + 3	3	42.86	3	37.50	2	66.67
3 + 4	1	14.29	3	37.50		
4 + 3			1	12.50		
4 + 4	2	28.57	1	12.50	1	33.33
5 + 5	1	14.29				
Positive Nodes						
1–3	5	71.43				
4–6	1	14.29				
>7	1	14.29				

**Table 2:** Clinical data from plasma samples used in microarrays.

Total of Samples **	Prostate Cancer		Healthy Donors
	*n* = 16		*n* = 10
Age			
Mean	75.06		28.1
Range	55–93		22–45
PSA (ng/mL)			
Mean	84.72		-
Range	2.74–566		-
Gleason Score *	*n*	%	
3 + 3	4	57.14	
3 + 4	2	28.57	
4 + 3	1	14.29	
4 + 4	3	42.86	
4 + 5	1	14.29	
5 + 4	1	14.29	
5 + 5	2	28.57	
TNM Stage	*n*	%	
T1A	1	6.25	
T2A	9	56.25	
T2B	3	18.75	
T2C	3	18.75	

* No Gleason score was reported for two samples. ** None of the patients presented lymph node invasion or disseminated metastasis at the time of diagnosis.

**Table 3:** Data of differential expression of miRNA in metastatic tissue and plasma.

Tissue	miRNA	Fold Change	*p*-Value
Metastasis vs. Tumor	hsa-miR-150-5p	30.81	0.003
	hsa-miR-23b-3p	−2.03	0.0472
	hsa-miR-140-3p	3.87	0.0213
Metastasis vs. Lymph Node	hsa-miR-150-5p	No significant differential expression	
	hsa-miR-23b-3p	2.36	0.0283
	hsa-miR-140-3p	No significant differential expression	
Tumor vs. Lymph Node	hsa-miR-150-5p	−23.14	0.0001
	hsa-miR-23b-3p	4.79	0.0009
	hsa-miR-140-3p	−3.8	0.0049
PCa vs. Healthy Plasma	hsa-miR-150-5p	−2.47	0.0162
	hsa-miR-23b-3p	−2.4	0.0308
	hsa-miR-140-3p	−1.63	0.0262

**Table 4:** Clinical data of plasma samples used in miR-150-5p and miR-23b-3p validation.

Total of Samples *	Prostate Cancer		Healthy Donors
	*n* = 31		*n* = 12
Age			
Mean	73.35		35.25
Range	57–86		26–45
PSA (ng/mL)			
Mean	16.709		-
Range	1.99–87.6		-
Gleason Score	*n*	%	
3 + 3	8	25.81	
3 + 4	7	22.58	
4 + 3	8	25.81	
4 + 4	4	12.90	
4 + 5	3	9.68	
5 + 5	1	3.23	

* None of the patients presented metastasis at the time of the sample collection.

**Table 5:** Clinical data from tissue samples used in miR-150-5p second validation.

	Primary Tumor Tissue		Metastatic Lymph Node Tissue			Normal Prostate Tissue
Total of samples	*n* = 15		*n* = 5			*n* = 20
Age						
Mean	75.46		70.2			25.75
Range	51–90		62–78			22–30
PSA (ng/mL)						
Mean	33.2		-			-
Range	0.7–106		-			-
Gleason Score *	*n*	%	Gleason Score	*n*	%	
3 + 4	4	26.67	3 + 4	-	-	
4 + 3	3	20	4 + 3	-	-	
4 + 4	-	-	4 + 4	1	20	
4 + 5	3	20	4 + 5	2	40	
5 + 4	3	20	5 + 4	2	40	
5 + 5	1	6.67	5 + 5	-	-	
Stage **	*n*	%	Number of Positive LNs			
IIB	2	13.33	2	1	20	
IIIB	2	13.33	4	1	20	
IIC	1	6.67	6	2	40	
IIIC	4	26.67	7	1	20	
IV	3	20				

* No Gleason score was reported for one sample of primary tumor. ** No tumoral stage was reported for two samples of primary tumor.

## Data Availability

Not applicable.

## Supplementary Materials

The following supporting information can be downloaded at: https://www.mdpi.com/article/10.3390/cancers15164148/s1, Table S1: Differentially expressed miRNA between Metastatic Lymph Node and Tumor samples. Table S2: Differentially expressed miRNA between Metastatic Lymph Node and Lymph Node samples. Table S3: Differentially expressed miRNA between Tumor and Lymph Node samples. Table S4: Differentially expressed miRNA between PCa and Healthy plasma samples. Figure S1: Electron microscopy images from extracellular vesicles obtained from PCa cell lines. Figure S2: Expression of exosome protein markers in EVs isolated from PCa cell lines. Figure S3: Histograms of comparison of the total isolated EVs using ultracentrifugation and ATPS methods. Figure S4: Comparison of total EVs/uL positive for tetraspanins and exosomes between ultracentrifugation and ATPS. Figure S5: Histograms of tetraspanin positivity of PCa plasma EVs samples. Figure S6: Cytometry analysis of plasma EVs’ tetraspanin expression. Figure S7: Percentage of EVs positive for tetraspanins in cell lines versus plasma samples. Figure S8: Distribution of events/uL and exosomes by nanoscale cytometry in different cell lines. Table S5: Genes related to each GO term from the potential targets of miR-150-5p.
