# Supplementary material for: Unraveling the Role of EV-Derived miR-150-5p in Prostate Cancer Metastasis and Its Association with High-Grade Gleason Scores: Implications for Diagnosis
Source: Cancers (Basel). 2023 Aug 17;15(16):4148. doi: 10.3390/cancers15164148 (PMC10453180; doi:10.3390/cancers15164148)
Supplement: Supplementary file 1 [file cancers-15-04148-s001.zip › 2Supplementary Material NLC paper 2023.pdf]

## Supplementary Materials

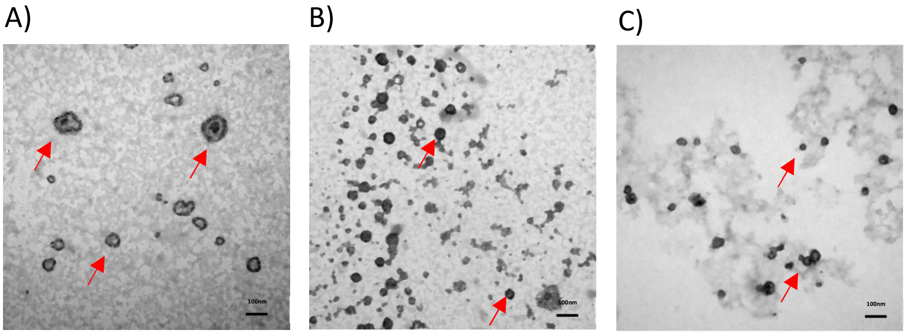


**Figure S1. Electron microscopy images from extracellular vesicles obtained from PCa cell lines.** Extracellular vesicles were isolated by ultracentrifugation from PCa cell lines. The image shows EVs with predominant circular shapes and well-defined borders. A) LNCaP EVs B) PC3 EVs. C) DU145 EVs. All images were taken at 100,000X.

LNCaP cells produced the largest EVs (60-130nm) with a bi-concave shape and a well-defined membrane (Figure S1A). PC3 cells produced EVs ranging in size from 45-58nm with well-defined edges and a circular shape (Figure S1B), while DU145 cells produced the smallest EVs (25-60nm) with a double membrane that was visible (Figure S1C).

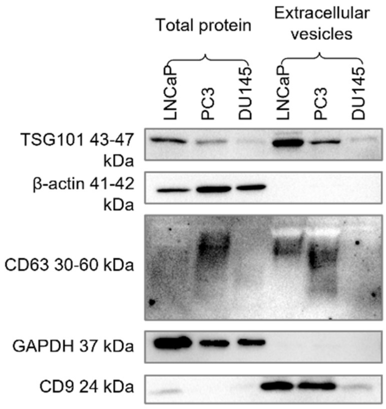


**Figure S2. Expression of exosome protein markers in EVs isolated from PCa cell lines.** TSG101 expression is stronger in EVs samples than in total protein. CD9 is only present in EVs samples. CD63 is detected in total protein of PC3 cells, its expression **i**s increased in vesicles. Negative controls β-Actine and GAPDH, are only present in total protein.

To confirm the presence of extracellular vesicles in the samples, western blot analysis was performed to determine the exosome markers TSG101, CD63, and CD9 (Figure S2). In addition, negative exosome markers such as GAPDH and β-Actin were used to ensure the absence of intracellular markers in the EV samples.

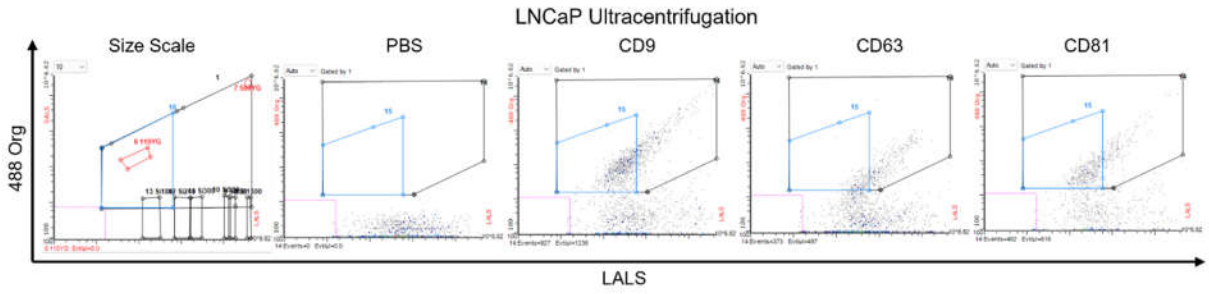
LNCaP Ultracentrifugation
Size Scale
PBS
CD9
CD63
CD81
488 Org
LALS

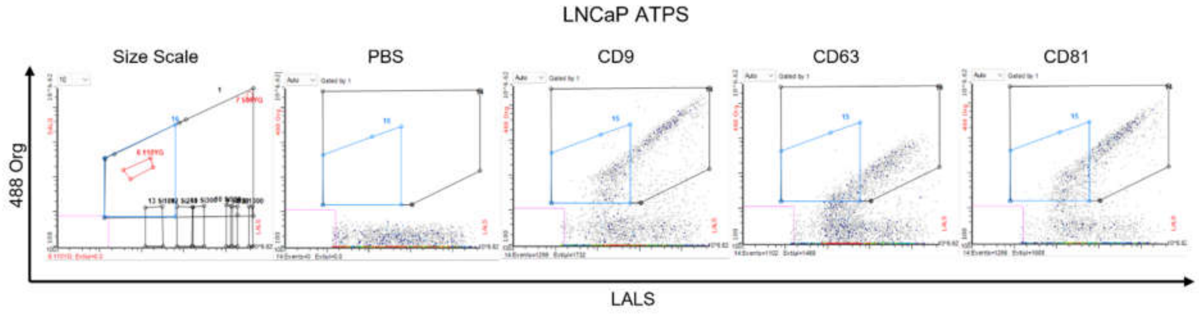
LNCaP ATPS
Size Scale
PBS
CD9
CD63
CD81
488 Org
LALS

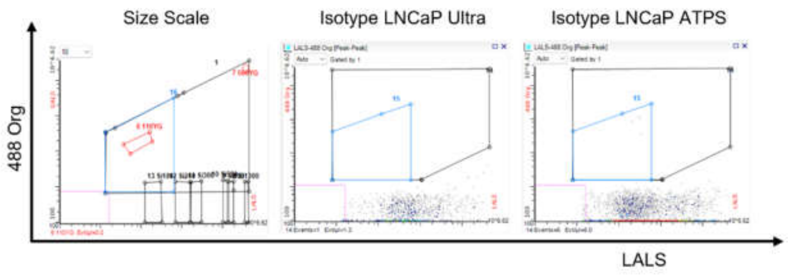
Size Scale
Isotype LNCaP Ultra
Isotype LNCaP ATPS
488 Org
LALS

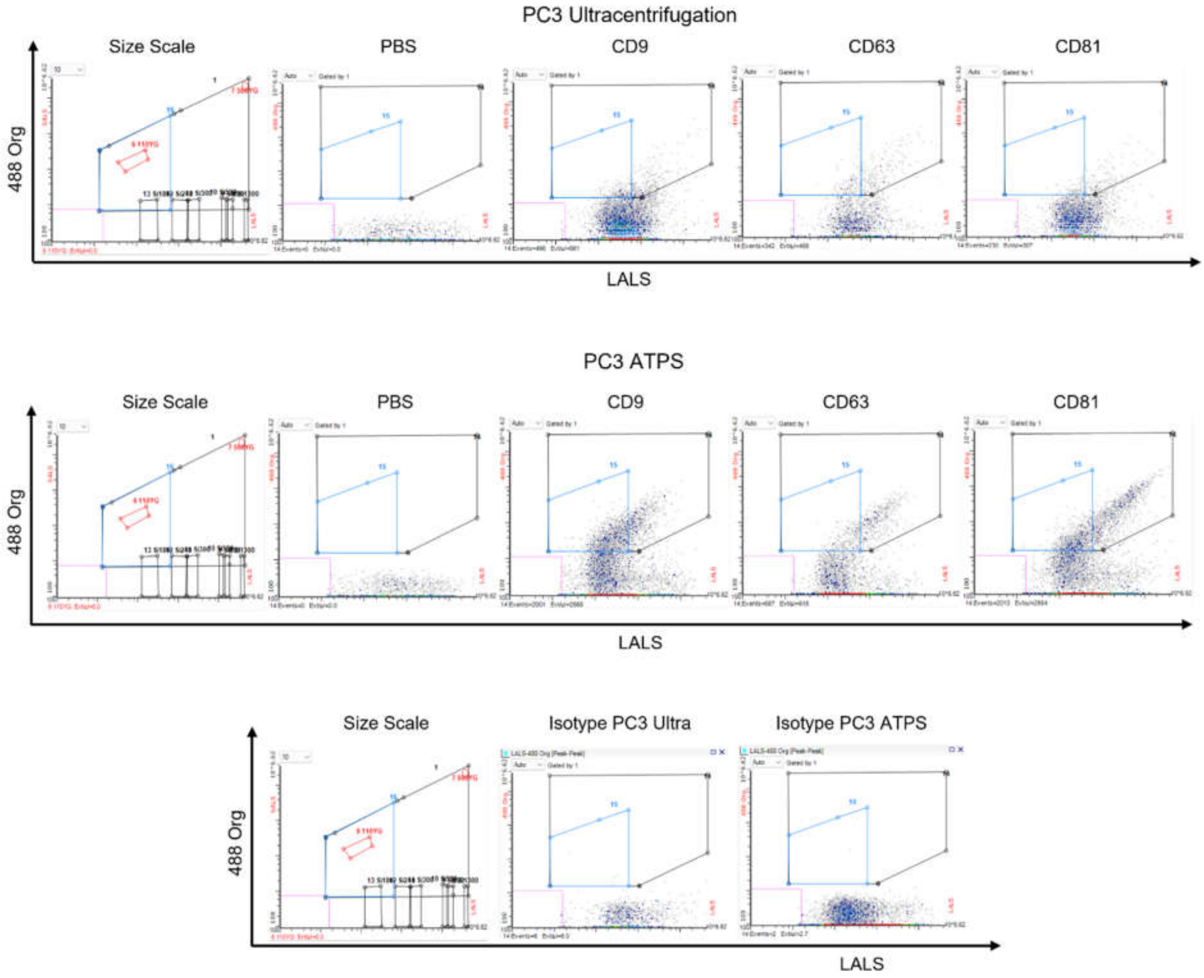
PC3 Ultracentrifugation
Size Scale
PBS
CD9
CD63
CD81
488 Org
LALS
PC3 ATPS
Size Scale
PBS
CD9
CD63
CD81
488 Org
LALS
Size Scale
Isotype PC3 Ultra
Isotype PC3 ATPS
488 Org
LALS

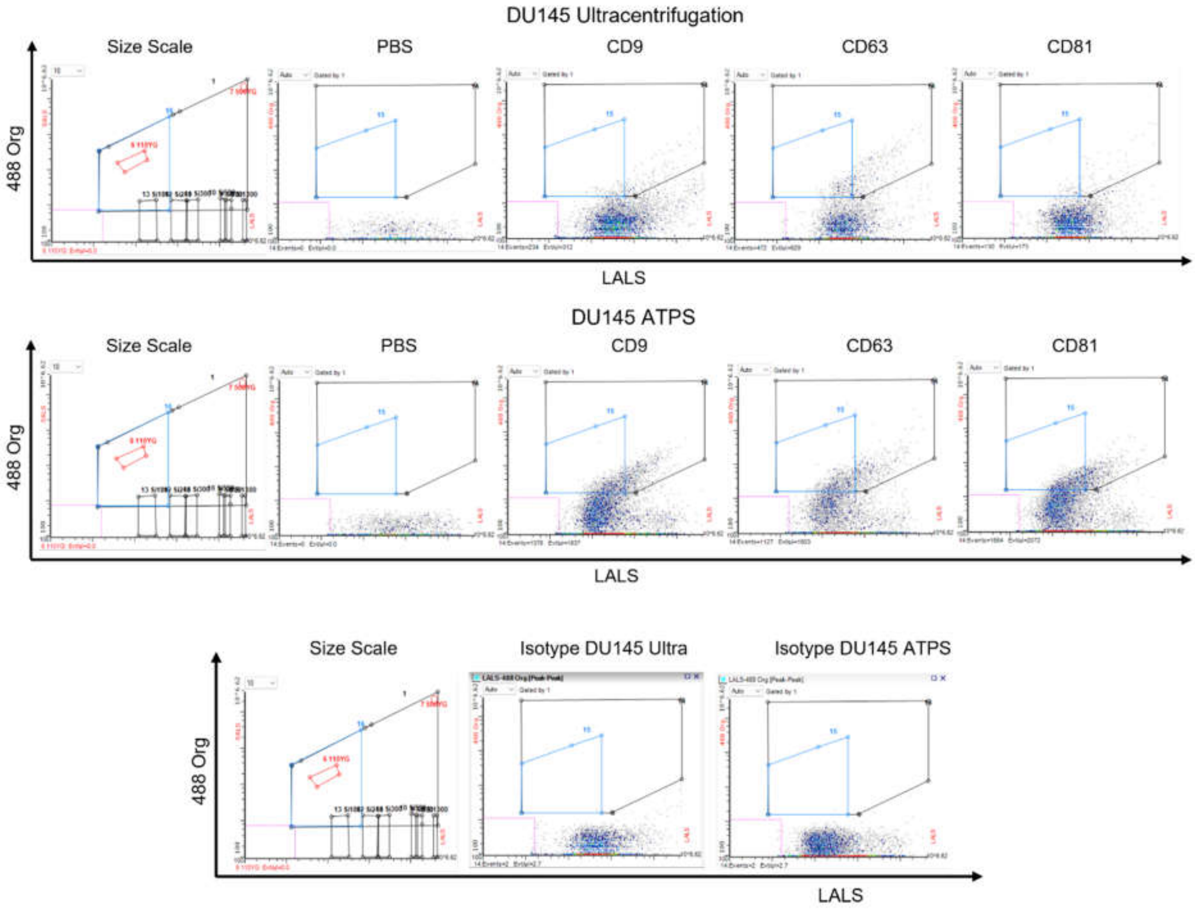


**Figure S3. Histograms of comparison of the total isolated EVs using ultracentrifugation and ATPS methods.** The histograms illustrate the number of EVs positive for CD9, CD63 and CD81 in different cell lines with ATPS and ultracentrifugation**.** The first image in each histogram illustrates the size scale, which was established by measuring magnetic beads of different sizes: 180, 240, 300, 590, 880 and 1300 nm. The blue gate encompasses particles with a size smaller than 200nm according to the magnetic beads scale. The isotype controls for each cell line are shown, all of them are clean with no significant events registered.

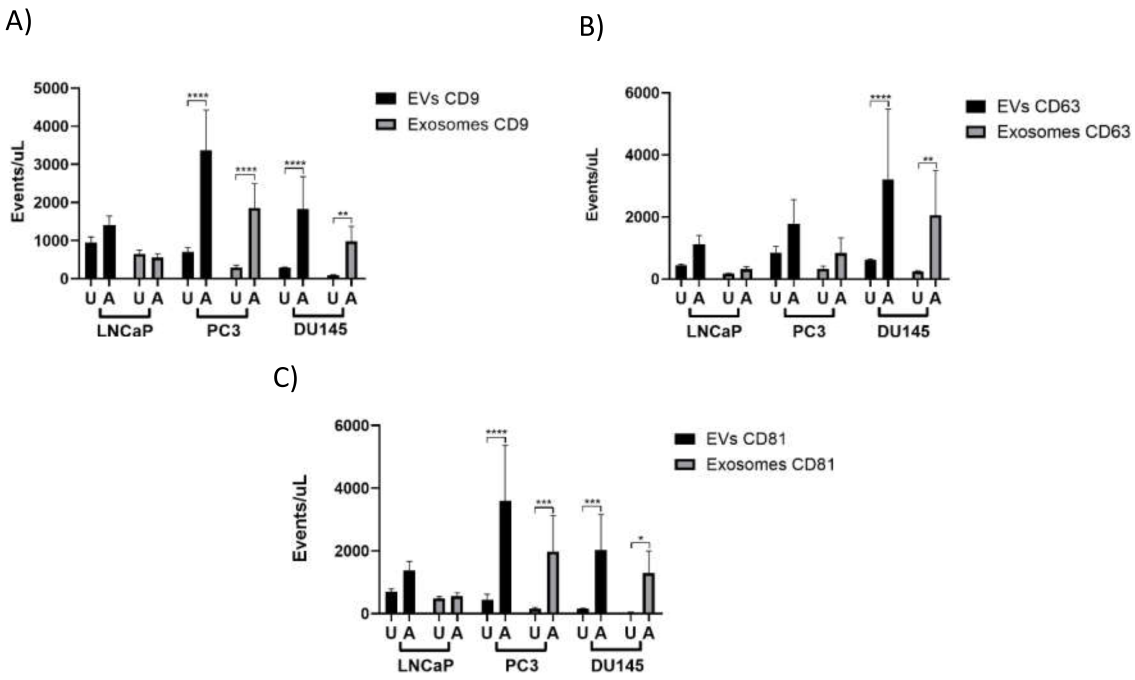


**Figure S4. Comparison of total EVs/uL positive to tetraspanins and exosomes between ultracentrifugation and ATPS.** A) CD9, B) CD63, C) CD81. The graphs display the event count per microliter obtained by ultracentrifugation (U) and ATPS (A) from different cell lines. *p<0.05, **p<0.01***p<0.001, ****p<0.0001

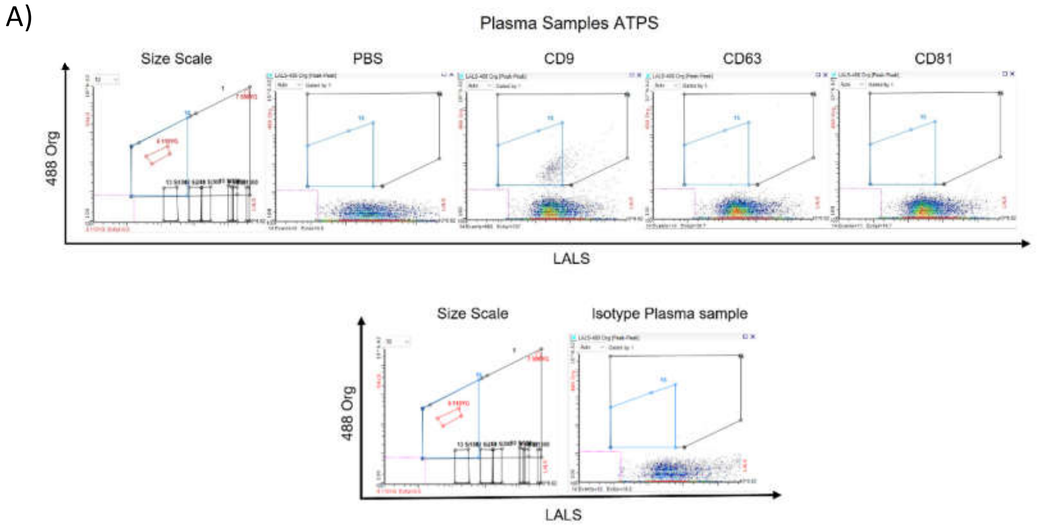


**Figure S5. Histograms of tetraspanins positivity of PCa plasma EVs samples.** The histograms illustrate the number of EVs positive for CD9, CD63 and CD81 in a representative PCa plasma EV sample. The first image in each histogram illustrates the size scale, which was established by measuring magnetic beads of different sizes:

180, 240, 300, 590, 880 and 1300 nm. The blue gate encompasses particles with a size smaller than 200nm according to the magnetic beads scale. The isotype controls is clean with no significant events registered.

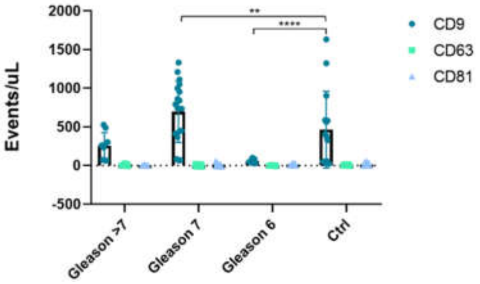


**Figure S6. Cytometry analysis of plasma EVs tetraspanins expression.** Counting of EVs/uL positive to tetraspanins according to Gleason score. EVs are enriched in CD9. Significant differences have been found in Gleason7 compared to control samples. **ANOVA two wat, Dunnett test. *<0.05, **<0.01, ****<0.0001.**

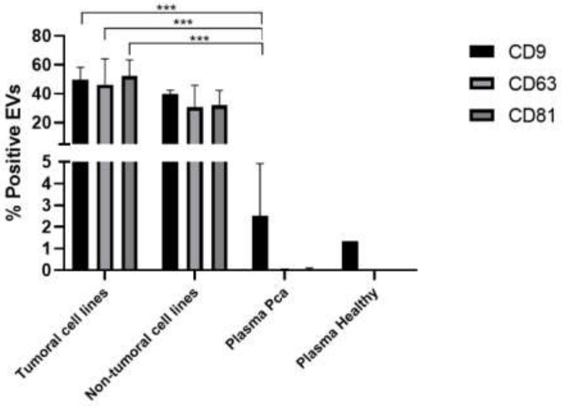


**Figure S7. Percentage of positive EVs to tetraspanins in cell lines versus plasma samples.** The graph shows the percentage of vesicles obtained from cell lines is significantly higher compared to those obtained from plasma samples. *p<0.05

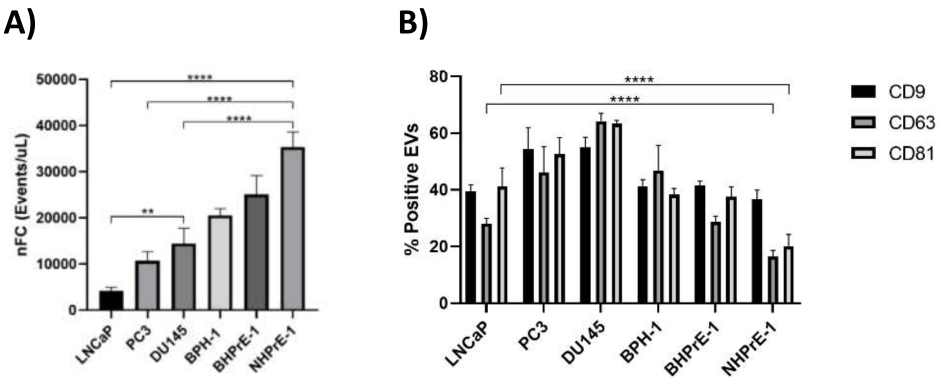


**Figure S8. Distribution of events/uL and exosomes by the nano scale cytometry in different cell lines. A)** Shows the total events/uL registered in each cell line, non-tumoral cells show the highest number of events. **B)** Shows the percentage of exosomes registered in each cell line. Tumoral cell lines show higher number of exosomes CD63 and CD81postive, than non-tumoral cells. *p<0.05
